# State-Dependent Propagation of Neuronal Sub-Population in Spontaneous Synchronized Bursts

**DOI:** 10.3389/fnsys.2016.00028

**Published:** 2016-03-31

**Authors:** Yuichiro Yada, Ryohei Kanzaki, Hirokazu Takahashi

**Affiliations:** ^1^Research Center for Advanced Science and Technology, The University of TokyoTokyo, Japan; ^2^Department of Mechano-Informatics, Graduate School of Information Science and Technology, The University of TokyoTokyo, Japan; ^3^Japan Society for the Promotion of ScienceTokyo, Japan

**Keywords:** spontaneous synchronized burst, state-dependent activity, microelectrode array, dissociated culture, metastable dynamics

## Abstract

Repeating stable spatiotemporal patterns emerge in synchronized spontaneous activity in neuronal networks. The repertoire of such patterns can serve as memory, or a reservoir of information, in a neuronal network; moreover, the variety of patterns may represent the network memory capacity. However, a neuronal substrate for producing a repertoire of patterns in synchronization remains elusive. We herein hypothesize that state-dependent propagation of a neuronal sub-population is the key mechanism. By combining high-resolution measurement with a 4096-channel complementary metal-oxide semiconductor (CMOS) microelectrode array (MEA) and dimensionality reduction with non-negative matrix factorization (NMF), we investigated synchronized bursts of dissociated rat cortical neurons at approximately 3 weeks *in vitro*. We found that bursts had a repertoire of repeating spatiotemporal patterns, and different patterns shared a partially similar sequence of sub-population, supporting the idea of sequential structure of neuronal sub-populations during synchronized activity. We additionally found that similar spatiotemporal patterns tended to appear successively and periodically, suggesting a state-dependent fluctuation of propagation, which has been overlooked in existing literature. Thus, such a state-dependent property within the sequential sub-population structure is a plausible neural substrate for performing a repertoire of stable patterns during synchronized activity.

## Introduction

Repeating stable spatiotemporal patterns emerge in synchronized spontaneous activity *in vivo* (Lee and Wilson, 2002; Ji and Wilson, 2007; Luczak et al., 2007; Villette et al., 2015), *in vitro* (Beggs and Plenz, 2003, 2004; Ikegaya et al., 2004), and in dissociated cultures (Segev et al., 2004; van Pelt et al., 2004; Eytan and Marom, 2006; Madhavan et al., 2007; Rolston et al., 2007; Schroeter et al., 2015). The repertoire of such patterns can serve as memory, or a reservoir (Maass et al., 2002; Sussillo and Abbott, 2009) of information, in a neuronal network; moreover, the variety of patterns may represent the memory capacity in the network (Shew et al., 2011). Furthermore, spatiotemporal patterns in spontaneous activity are often similar to those of evoked activity against external events (Arieli et al., 1996; Tsodyks et al., 1999; Kenet et al., 2003; Luczak et al., 2009), suggesting that the variety of spontaneous patterns constrain the processing capacity of external inputs (Luczak et al., 2009; Villette et al., 2015). Our present interest is therefore the neural mechanism required to build a repertoire of stable spatiotemporal patterns in synchronized spontaneous activities.

Both theoretical and experimental studies have demonstrated that stable patterns emerge in a sequential structure of a neuronal network, where each synaptic connection is unreliable, yet synchronized activities of a particular sub-population reliably elicit another sub-population activity (Abeles, 1991; Aertsen et al., 1996; Diesmann et al., 1999; Ikegaya et al., 2004). Nevertheless, the way in which the repertoire of patterns is built in such a sequential structure remains elusive.

To address this problem, state-dependency of neuronal activity is a plausible neural underpinning (Buonomano and Maass, 2009). Recently, cortical spontaneous activities were characterized as having multiple “metastable states” itinerating in an activity dependent manner (Mazzucato et al., 2015). A particular state should continue during a quiescent period (Dranias et al., 2013, 2015; Ju et al., 2015) because cellular and synaptic properties governing states are likely to last without explicit spiking (Buonomano and Maass, 2009). Based on these studies, we hypothesize that (i) stable spatiotemporal patterns in synchronized spontaneous activity are generated by sequential activation of sub-populations, and that (ii) these patterns are generated in a state-dependent manner, whereby multiple metastable states can be defined as a finite continuous period.

In the present study, we test our hypotheses in dissociated neuronal cultures. To date, spontaneous activities in neuronal cultures have been well characterized with a microelectrode array (MEA) (Beggs and Plenz, 2004; Eytan and Marom, 2006; Madhavan et al., 2007). However, the spatial resolution of conventional MEA is insufficient to capture the whole activity in the neuronal network, potentially causing misestimation of population properties (Gerhard et al., 2011; Ribeiro et al., 2014). To overcome this technical pitfall, we use cutting-edge complementary metal-oxide semiconductor (CMOS) microelectrode arrays (MEAs) (Berdondini et al., 2009; Frey et al., 2010; Obien et al., 2014; Müller et al., 2015), which offer excellent spatiotemporal resolution for investigating neuronal networks *in vitro* (Gandolfo et al., 2010; Bakkum et al., 2013a; Panas et al., 2015). The CMOS MEA used in this study can simultaneously measure neural activities from 4096 sites within 2.67 × 2.67 mm^2^ at a sampling rate of 7 kHz. The high-dimensional spatiotemporal activity patterns are then characterized by non-negative matrix factorization (NMF) (Lee and Seung, 1999; Leonard et al., 2015; Wei et al., 2015) in order to visualize whether and how sub-populations are sequentially activated in a state-dependent manner.

We demonstrate that cultured neurons obviously perform a repertoire of multiple spatiotemporal patterns in spontaneous synchronized activity, while different patterns share a partially similar sequence of sub-populations. This supports the concept that the network has invariant sequential structures of sub-populations. Additionally, similar spatiotemporal patterns appear consecutively, which suggests that pattern generation is state-dependent. Our experimental results provide compelling evidence that a repertoire of stable neural patterns is generated in a state-dependent manner.

## Materials and methods

### Cell culture

All experimental protocols were approved by the ethical committee of the University of Tokyo and conducted in accordance with the “Guiding Principles for the Care and Use of Animals in the Field of Physiological Science” by the Japanese Physiological Society. The cell culture procedure was based on previous reports (Bakkum et al., 2013a) and was slightly modified. Cortices were dissected from E18 Wistar rats and dissociated by 0.25% trypsin-EDTA (Invitrogen) and trituration. For cell adhesion, the electrode area of the high-density CMOS MEA (3Brain, Biochip 4096S) was coated with a 20-ul drop of 0.05% polyethylenimine (Sigma) and then a 20-ul drop of 0.02 mg/ml laminin (Sigma). On the MEAs, 30,000–40,000 cells were seeded with cell plating media: 850 ul of NeuroBasal (Invitrogen) supplemented with 10% horse serum (HyClone), 2% B27 (Invitrogen), and 0.5 mM GlutaMAX (Invitrogen). After 24 h, the media were replaced with cell growth media: 850 ul of DMEM (Invitrogen) supplemented with 10% horse serum, 0.5 mM GlutaMAX, and 10 ug of sodium pyruvate. Cultures were maintained in an incubator at 37°C and 5% CO_2_ humidified atmosphere. Half the media were exchanged twice a week. For avoidance of evaporation and infection, the well on the chip was covered with a custom-made lid except for the period during the medium exchange (Potter and DeMarse, 2001).

### Recording with high-density CMOS MEAs

Extracellular voltage was recorded using a commercialized high-density CMOS MEA system (3Brain). Biochip 4096S (3Brain) contains 4096 electrodes; the scale of the electrode is 21 × 21 um, and the distance of neighboring electrodes is also 21 um. The electrodes are squarely located in the 2.67 × 2.67 mm area. Extracellular signals were simultaneously captured from the 4096 electrodes through the CMOS MEA interface, BioCAM4096 (3Brain), at a sampling rate of 7 kHz. They were recorded using BrainWave (3Brain) computer software. Ten minutes of spontaneous activities of five cultures at approximately 21 days *in vitro* (DIV) were recorded; their spontaneous activities at approximately 10 DIV were also recorded for comparison. Recording was performed outside the incubator. The recording space was shielded with a blackout curtain to avoid potential effects of ambient light (Imfeld et al., 2008) and maintained at 35–36°C atmosphere.

### Spike detection

Spikes were detected from recorded data by using a precise spike timing detection (PTSD) algorithm (Maccione et al., 2009) installed in BrainWave. The parameters for the PTSD algorithm were as follows: standard deviation factor, 10.0; peak life-time period, 2.0 ms; refractory period, 1.0 ms. The timing of each spike was assigned to the timing of its negative peak. The median of the peak amplitude of detected spikes was calculated at each electrode; spikes detected at electrodes with lower median peaks than the threshold were excluded from the following process. The threshold was the third quartile of the median peaks, which was qualified by manual inspection. Spike sorting was not performed in this experiment.

### Burst detection

Synchronized bursts (Kamioka et al., 1996) were detected from recorded spontaneous activity by a slightly modified version of the existing adaptive algorithm (Bakkum et al., 2013b). If *N*_*spike*_ spikes occurred at all electrodes in total within less than *T* ms, the period was defined as a burst. The threshold time, *T*, is adaptively determined from the inter-spike interval (ISI) of each culture. The distribution of ISI typically forms bimodal shapes; an interval that takes the minima at the valley of the distribution is chosen as *T*. Here, we set *N*_*spike*_ as 200 because the number of recording channels was larger than the setup used in the original paper. Additionally, *post-hoc* processing was conducted for avoidance of burst fragmentation. The original method has excellent sensitivity in detecting small sizes of bursts; however, it separated a large burst into several small bursts in some cases. Thus, if an interval between two consecutive bursts was less than 100 ms, the two bursts were merged into a single burst.

### Evaluation of burst peak amplitude distributions

The maximum number of array-wide spikes in a 10-ms time bin during a burst was defined as the peak amplitude of the burst. Distribution of spontaneous burst peak amplitude was evaluated to check diversity of bursts. The distribution of bursts in dense-plated cultures showed fixed-peak-amplitude bursts or “super bursts” at approximately 10 DIV, while they showed bimodal or long-tailed ones at approximately 20 DIV (Wagenaar et al., 2006b). Kurtosis of the distribution,

$$k=\frac{E[{\left(x-E[x]\right)}^{4}]}{E{\left[{\left(x-E[x]\right)}^{2}\right]}^{2}}\;-3,$$

where *x* is the value obeying the distribution, and the chi-square goodness-of-fit test for normal distribution, were used to evaluate unimodality of the distributions.

### Sub-population pattern extraction by non-negative matrix factorization (NMF)

The number of spikes occurred in 10-ms time bins were counted at all electrodes and a 4096 × 60,000 matrix was obtained. The matrix was defined as an observed matrix, *Y*. The width of the bin was chosen on the basis of the time step used in the previous report that observed multiple recursive spatiotemporal patterns in synchronized bursts (Madhavan et al., 2007). It was hypothesized that a part of the neurons in a network constitute a co-active sub-population in the temporal resolution; the sequential activation of such sub-neuronal populations generates repeatable spatiotemporal activity of neurons as synchronized bursts. In this model, the activities of sub-populations are captured as reproducible spatial patterns; we refer to them as sub-population patterns (SPPs).

It was assumed that each element of the observation matrix, *y*_*i, t*_(*i* = 1, 2, …, 4096; *t* = 1, 2, …, 60000), was sampled from the Poisson process with parameter s_i, t_, which indicates an instantaneous firing rate,

$$\begin{array}{l} p\left(y_{i,t}\right)=Poisson\left(y_{i,t}|s_{i,t}\right). \end{array}$$

It was additionally assumed that the firing rate of all 4096 electrodes at each bin was generated by a linear combination of *D* pieces of SPPs, where *D* is the dimension of network activity. Consequently, the instantaneous firing rate matrix, *S* (4096 × 60,000 matrix), was represented as the product of an SPP matrix, *H* (4096 × *D* matrix), which contains an SPP at each column, and a sub-population activation weight (SPAW) matrix, *W* (*D*×60, 000 matrix), which contains the coefficients for linear combination,

$$\begin{array}{l} S\;=\;H\times \;W. \end{array}$$

Notably, the SPPs could have overlapping electrodes in this model. The element of the observed matrix is the number of spikes and is thus non-negative. We thus define elements of the SPP matrix and SPAW matrix as also being non-negative. With this assumption, the SPP and SPAW matrices can be derived from the observed matrix using NMF (Lee and Seung, 1999). NMF with the generalized Kullback–Leibler divergence cost function actually assumes the Poisson generative model described above. Thus, we implemented the following optimization:

$$\begin{array}{l} minimize\;D_{KL}\;\left(Y|HW\right)\\ s.t.\forall i,\forall d,h_{i,d}\;\geq \;0;\forall d,\forall t,w_{d,t}\;\geq \;0, \end{array}$$

where generalized Kullback–Leibler divergence *D*_*KL*_ between matrices is:

$$\begin{array}{l} D_{KL}\left(A|B\right)\;=\;\underset{m,n}{\sum }\left(A_{m,n}log\frac{A_{m,n}}{B_{m,n}}\;-\;A_{m,n}\;+\;B_{m,n}\right). \end{array}$$

The algorithm proposed by Lee and Seung (2001) was adopted to solve this problem. Open source MATLAB codes were used with slight modification^1^. The number of SPPs, *D*, was empirically determined to be 10 (*d* = 1, 2, …, 10). The initial values of the matrix elements were randomly set, and the iteration loop was implanted 500 times. The calculation was independently repeated ten times. The result with the minimum cost function among all trials was used for further analysis.

### Burst pattern classification

Bursts were classified into several classes to identify multiple recursive spatiotemporal patterns. In the present study, SPAWs during a bursting period were used as a burst feature matrix (BFM) to characterize the burst. First, however, time spans of bursts were adjusted from detected burst periods to compare spatiotemporal patterns. The initiation point of a burst was defined as the first bin where ten spikes or more were observed in the whole network in a detected burst period.

All BFMs have the same length of time before the initiation points (pre-initiation length) and the same length of time after initiation pointsı (post-initiation length). Thus, the pre-initiation length was determined to be 100 ms to avoid inclusion of previous bursts in a BFM. The post-initiation length was adjusted depending on the cultures to include the burst with the longest length from its initiation point. Then, BFMs were classified by correlation-based hierarchical clustering with some modifications from previous studies. The maximum peak of cross correlation was defined as the similarity between BFM *A* and BFM *B* to avoid the effects of the extraction of burst periods.

$$\begin{array}{l} Corr\left(A,A\right)\;=\;\overset{L}{\underset{l=1}{\sum }}\overset{D}{\underset{d=1}{\sum }}A_{d,l}A_{d,l} \end{array}$$

$$\begin{array}{l} Similarity\left(A,\;B\right)\;=\;\underset{{}_{k}}{\max}\sum_{l=1}^{L}\sum_{d=1}^{D}\frac{A_{d,l}}{Corr{\left(A,A\right)}^{1/2}}\frac{B_{d,l-k}}{Corr{\left(B,B\right)}^{1/2}}\\ \;\left(k=-L,\;L\;+\;1,\ldots .,0,\ldots ,L-1,L\right), \end{array}$$

where *L* is the length of the BFMs, and zero is inserted into the elements of BFMs if *l* − *k* < 0 or *l* − *k* > *L*. If BFM *A* and BFM *B* are identical, *Similarity*(*A, B*) = 1. Similarities between all pairs of the BFMs were calculated; the highest similar pair was grouped. An averaged BFM of the group was then used as a new BFM that represents the grouped bursts.

The above procedure was repeated until all BFMs were merged into one class group. Subsequently, the number of classes that maximize the contrast function (Beggs and Plenz, 2004) was determined as the optimal number of classes. However, if the largest class occupied more than 90% of all bursts, the next peak of the contrast function was selected as the number of the optimal class because misdetected bursts could form a small fraction in some cases.

### Sub-population sequence analysis

Sequences of sub-population activity between different classes of bursts were compared. The comparison was conducted because the sequences are assumed to be partially invariant regardless of the classes of overall spatiotemporal patterns if there exists stable sequential propagation between sub-populations. First, five of the ten sub-populations with the largest peaks of SPAWs in the averaged burst were selected. The other five small SPAWs were excluded as burst-unrelated, or low contributing sub-populations. Then, spatiotemporal patterns of bursts were converted into a sequence of the timing when each sub-population took the maximum SPAWs. One of the burst classes with the largest summation of SPAWs was defined as a template class. Moreover, the sub-population sequence calculated from the averaged template-class bursts was defined as a template sequence.

Here, partial similarity with respect to the template sequence was evaluated for (i) sub-population sequences in template classes, (ii) those in non-template classes, and (iii) randomized sequences. The partial similarity was measured according to two kinds of criteria, which were adopted with slight modification from a previous study of memory replay in the hippocampus (Lee and Wilson, 2002). The first criterion is the number of times of permutation of a sub-population pair order to perfectly match the template sequence. The proportion of sub-population sequences that can match the template with the same or less than N permutation was used as the index of similarity, where N is a threshold value. The second criterion is the reproducibility of the sub-population pair order (duplet) or the sub-population trio order (triplet). A pair and a trio with the highest order consistency in all synchronized bursts were used as the duplet and triplet. The probability that the duplet or triplet was replayed within sequences was used as the index of similarity. Significance of partial similarity was statistically tested according to the similarity indices of actual sub-population sequences against those of randomized sequences. The number of randomized sequences was identical to the number of total (both the template class and non-template class) bursts. The significance was evaluated by the Mann–Whitney *U*-test.

### Sequence randomization

In sub-population sequence analysis, as well as in burst class consecutiveness analysis, randomized sequences were used to test the statistical significance of actual data. Random real numbers between [0 1] were sampled from the uniform distribution and assigned to all elements of the sequence. The elements of the sequence were then sorted according to the assigned numbers, and this sorted sequence was compared with actual data.

### Evaluation of burst class consecutiveness

Consecutiveness in sequences of burst classes was evaluated by probability of burst class transition. The probability that the same burst class was generated successively in actual data was compared with that of randomized burst-class sequences. One hundred randomized sequences were generated against each culture. The statistical significance of actual data was evaluated for each data, thereby testing the null hypothesis that the median of the probabilities in the randomized sequences is equal to the probability of the actual sequence by the one-sample Wilcoxon signed-rank test.

### Evaluation of periodical similarity in burst patterns

To evaluate the periodical appearance of spatiotemporal patterns in synchronized bursts, Fisher's g-statistic was used to test the significance of periodicity (Wichert et al., 2003). Fisher's g-statistic is defined as:

$$g=\frac{max_{i}I\left(\omega_{i}\right)}{\sum_{i=1}^{\left[N_{sample}/2\right]}I\left(\omega_{i}\right)},$$

where *I*(ω_*i*_) is the periodogram of the signal to evaluate, *N*_*sample*_ is the sample size of the signal, and ω_*i*_ is a discrete frequency of the signal, ω_*i*_ = 2π*i*∕*N*_*sample*_(*i* = 0, 1, 2, …, [*N*_*sample*_∕2]). The significance level of Fisher's g-statistic was determined from the distribution:

$$P(g>g^{*})\;=\;\sum_{m=1}^{M}{\left(-1\right)}_{\left[\frac{N_{sample}}{2}\right]}^{m-1}C_{m}{\left(1\;-\;mg^{*}\right)}^{\left([N_{sample}/2]-1\right)},$$

where *M* is the largest integer less than 1∕g^*^. Mean burst similarity according to (i) the difference of burst indices, and (ii) the difference of burst appearance time, from which linear components were subtracted, were used as *I*(ω_*i*_) in this study. For avoiding misestimation from small samples, the differences of burst indices with more than 20 pair samples and pairs of bursts appeared within 400 s were used.

## Results

High-density CMOS MEAs captured spontaneous neuronal activity from five cortical networks. Ten-minute recording was performed in each culture in two developmental conditions: in a developed period–−20.8 ± 2.2 (mean ± SD) DIV—and in a juvenile period— 9.8 ± 0.8 DIV—for comparison. The number of available electrodes that detected action potentials with amplitude larger than the threshold was typically around 1000 channels. Synchronized bursts of cortical neurons were observed from the juvenile periods and stably persisted through development (Kamioka et al., 1996). The bursts were detected with the adaptive algorithm (Bakkum et al., 2013b); the average number of detected bursts was 139 ± 68 (mean ± SD) in a developed period, whereas it was 88 ± 54 in a juvenile period.

**Figure 1:**
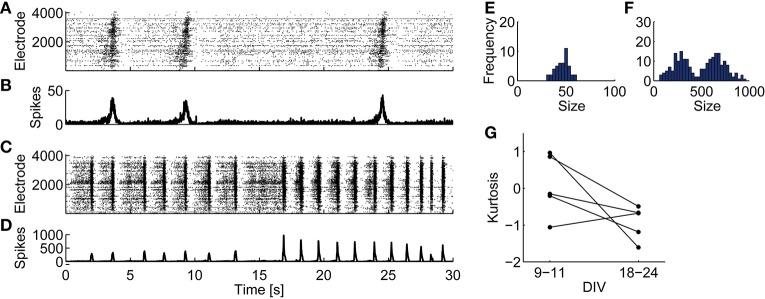
**Spontaneous spiking activities of cultured cortical neurons recorded on CMOS MEAs and distributions of burst peak amplitude**. **(A,B)** A representative raster plot **(A)** and the number of array-wide spikes **(B)** from 30 s of spontaneous activity recorded from a culture at 10 DIV. **(C,D)** A raster plot **(C)** and the number of array-wide spikes **(D)** from recorded data from a culture at 18 DIV. **(E)** Histogram of burst peak amplitude (the maximum number of array-wide spikes in 10-ms bins during synchronized bursts) from the same recorded data shown in **(A,B)**. **(F)** Histogram of burst peak amplitude from the same recorded data shown in **(C,D)**. **(G)** Kurtosis of the burst-peak-amplitude distribution from spontaneous activities at 9–11 DIV and those at 18–24 DIV.

Consistent with a previous study (Wagenaar et al., 2006b), juvenile cultures exhibited fixed-peak-amplitude bursts, while developed cultures exhibited variable-peak-amplitude bursts. Representative 30-s spontaneous activities of juvenile and developed cultures are shown in Figures 1A–D. A juvenile network shows homogeneous spatiotemporal spiking activity (Figures 1A,B). In a developed one, however, bursts show heterogeneous activity (Figures 1C,D). The distributions of burst peak amplitude—the maximum number of array-wide spikes in a 10-ms time bin during a bursting period—show a single peak in a juvenile culture (Figure 1E), but a bimodal shape in a developed culture (Figure 1F). Figure 1G shows kurtosis of the burst-peak-amplitude distributions. Kurtosis tends to drop and become apart from zero with development, except one culture (Culture #3), which was excluded from further analysis.

The chi-square goodness-of-fit test was performed for testing whether the burst-peak-amplitude distributions were represented with normal distribution. The developed-period distributions disobeyed normal distribution (Culture #1, *p* = 3.215 × 10^−8^, mean ± SD: 484.8 ± 225.1; Culture #2, *p* = 4.656 × 10^−10^, mean ± SD: 250.7 ± 176.2; Culture #4, *p* = 0.005357, mean ± SD: 198.7 ± 88.1; Culture #5, *p* = 3.775 × 10^−16^, mean ± SD: 1437 ± 631), while juvenile-periods ones were characterized with normal distributions (Culture #1, *p* = 0.7668, mean ± SD: 46.36 ± 6.92; Culture #2, *p* = 0.5175, mean ± SD: 46.65 ± 7.34; Culture #4, *p* = 0.9448, mean ± SD: 137.1 ± 41.5; Culture #5, *p* = 0.09618, mean ± SD: 91.02 ± 18.23). These results demonstrate that bursts in a developed period differ from those in a juvenile period in terms of characteristic peak amplitude. We thus hypothesize that the developed cultures recruit variable neuronal sub-populations to produce different patterns.

Spontaneous spiking activity of the cultured neurons was decomposed into SPPs and SPAWs. First, the frequency of spikes occurring in 10-ms bins were calculated at each electrode. Then, the NMF algorithm (Lee and Seung, 2001) decomposed a 4096-dimensional spike frequency matrix into ten SPPs, which represented the spatial patterns of reproducibly co-activated electrodes and ten-dimensional SPAWs. The number of spikes detected at each electrode at each time bin was modeled as generated by the Poisson process with a latent parameter, which corresponded to a firing probability at the time. The Kullback–Leibler divergence NMF hypothesizes that the latent parameters of the Poisson process are a linear combination of the SPPs.

**Figure 2:**
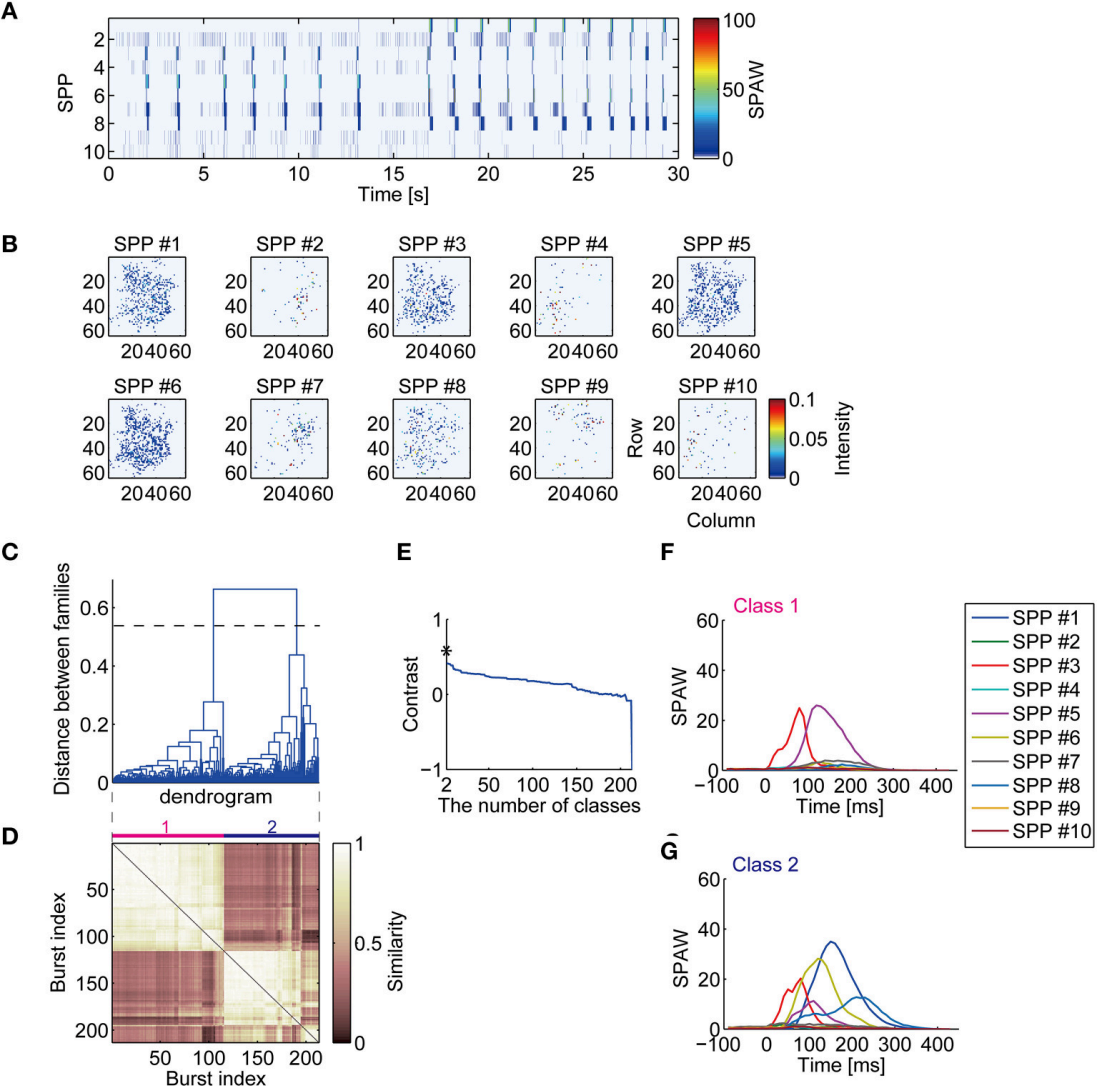
**Decomposition of high-dimensional neuronal activity and classification of synchronized burst patterns. (A)** Low-dimensional activity of the neuronal network represented with sub-population activation weights (SPAWs). The same period of Figures 1C,D is illustrated. **(B)** Sub-population patterns (SPPs) of spontaneous activity of cultured cortical neurons obtained with NMF. The SPPs are shown as corresponding to recording electrodes configuration. **(C)** A dendrogram represents a process of hierarchical grouping of BFMs. The dotted horizontal line indicates a selected level of the grouping. **(D)** A similarity matrix of sub-population activation weights during synchronized bursts. **(E)** A contrast function for the dendrogram shown in **(C)**. Asterisk indicates a maximum peak of the function. **(F**,**G)** Mean SPAWs within classified burst classes.

Non-monotonic repeating spatiotemporal patterns of synchronized bursts were observed from dimension-reduced activity obtained by NMF. The representative data in Figure 1A are decomposed into the temporal pattern of SPAW (Figure 2A) and SPPs (Figure 2B). The spatial distributions of co-activated electrodes in the SPPs did not always localized; some sub-populations had spatially localized activity patterns, while others had rather dispersed distributions, as illustrated in Figure 2B. Overall SPAW confirmed that synchronized burst patterns were reproducible both spatially and temporally. This finding is consistent with the previous study that demonstrated the stability of synchronized burst patterns in dissociated cultures (Eytan and Marom, 2006). However, the bursts in the first half (before 15 s) and those in the second half in Figure 2A appear to have different spatiotemporal patterns. For example, SPP #1 was recruited in the second half, but not in the first. Nevertheless, other SPPs seemed to be activated similarly in all synchronized bursts.

Spatiotemporal patterns of bursts were hierarchically clustered according to similarity of BFM and classified into several classes, as shown in the dendrogram in Figure 2C. The similarity matrix of bursts in Figure 2D, where indices of bursts are sorted according to the dendrogram in Figure 2C, suggests that temporal activation patterns of sub-populations in bursts are repeated. The number of classes was chosen to maximize the contrast function (Figure 2E) (Beggs and Plenz, 2004). The horizontal dotted line across the dendrogram in Figure 2C indicates the selected level of cut for the classes. Mean trajectories of SPAWs for each class are then illustrated in Figures 2F,G. Remarkably, temporal patterns of SPAWs in different classes seemed to be partially similar; spatiotemporal patterns in both classes were likely characterized as having common sub-populations with partially identical temporal orders. This suggests stable unidirectional propagation of sub-population across different classes. Such a sequential structure of neurons in spontaneous synchronized activity was suggested in previous reports (Eytan and Marom, 2006; Ham et al., 2008; Raichman and Ben-Jacob, 2008).

**Figure 3:**
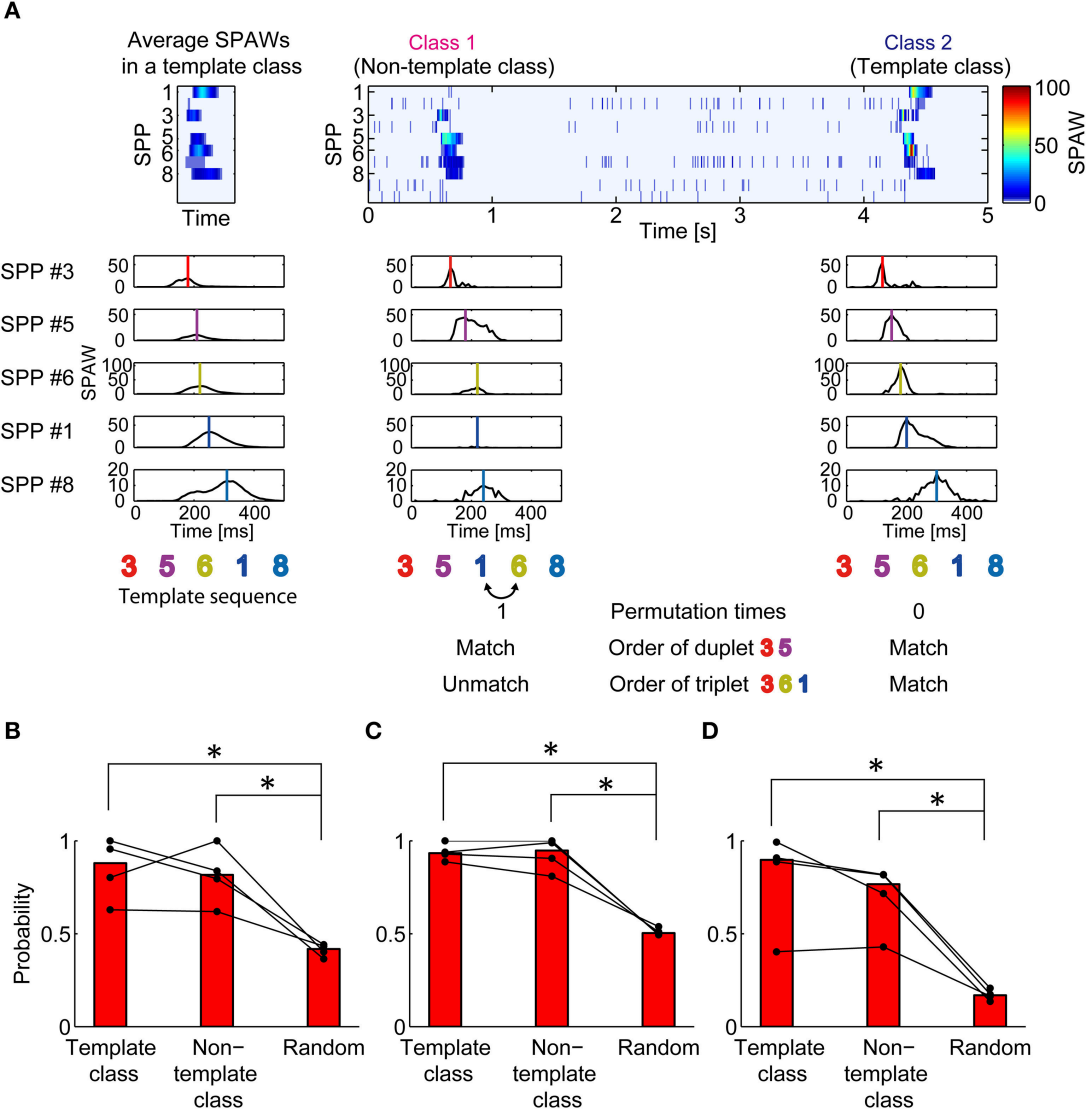
**Similarity in partial sequence of sub-population activation between synchronized burst classes. (A)** Illustration of the procedure to evaluate partial similarity between sub-population sequences of bursts. SPAWs during a burst were converted into a sequence of their peaks. Sub-population sequences of bursts were compared with the template sequence. Permutation times for matching refers to the way in which many pair permutations are required to match the template sequence. Duplet/triplet order matching indicates whether the order of two/three sub-populations matches the template sequence. **(B)** Probability that permutation times for matching is two or less. **(C,D)** Probability that the duplet **(C)** or the triplet **(D)** order matched the template. The most reproduced duplet or triplet was selected for analysis. The Mann–Whitney *U*-test. ^*^p < 0.05.

To evaluate partial similarity of spatiotemporal patterns among different classes, the sequences of sub-populations were compared. Figure 3A illustrates a schematic procedure of the analysis. Five out of ten sub-populations were selected according to the largest peaks of SPAWs in all averaged BFM to exclude effects from burst-unrelated or burst-less-related sub populations. Then, each burst was represented as a sequence of selected sub-populations. The burst class with the largest total SPAWs was defined as a template class; the sub-population sequence of averaged bursts in the template class was defined as a template sequence.

Figure 3B shows the probability that a sequence had partial similarity with the template sequence in the mean of pair permutation times. The threshold was set to two times here. Obviously, sequences in the template class had a higher probability of having partial similarity with the template sequence compared with randomly generated sequences (*p* = 0.02857 < 0.05). However, sequences in the non-template classes also showed higher probability than random sequences (*p* = 0.02857 < 0.05), although the median probability was slightly smaller than that of the template class. The findings in the permutation analysis were reconfirmed in the appearance probability of the same duplet/triplet order as shown in Figures 3C,D (*p* = 0.02857 < 0.05 in all comparisons). Thus, different spatiotemporal patterns in synchronized bursts were likely to have partially similar sequences of sub-populations.

**Figure 4:**
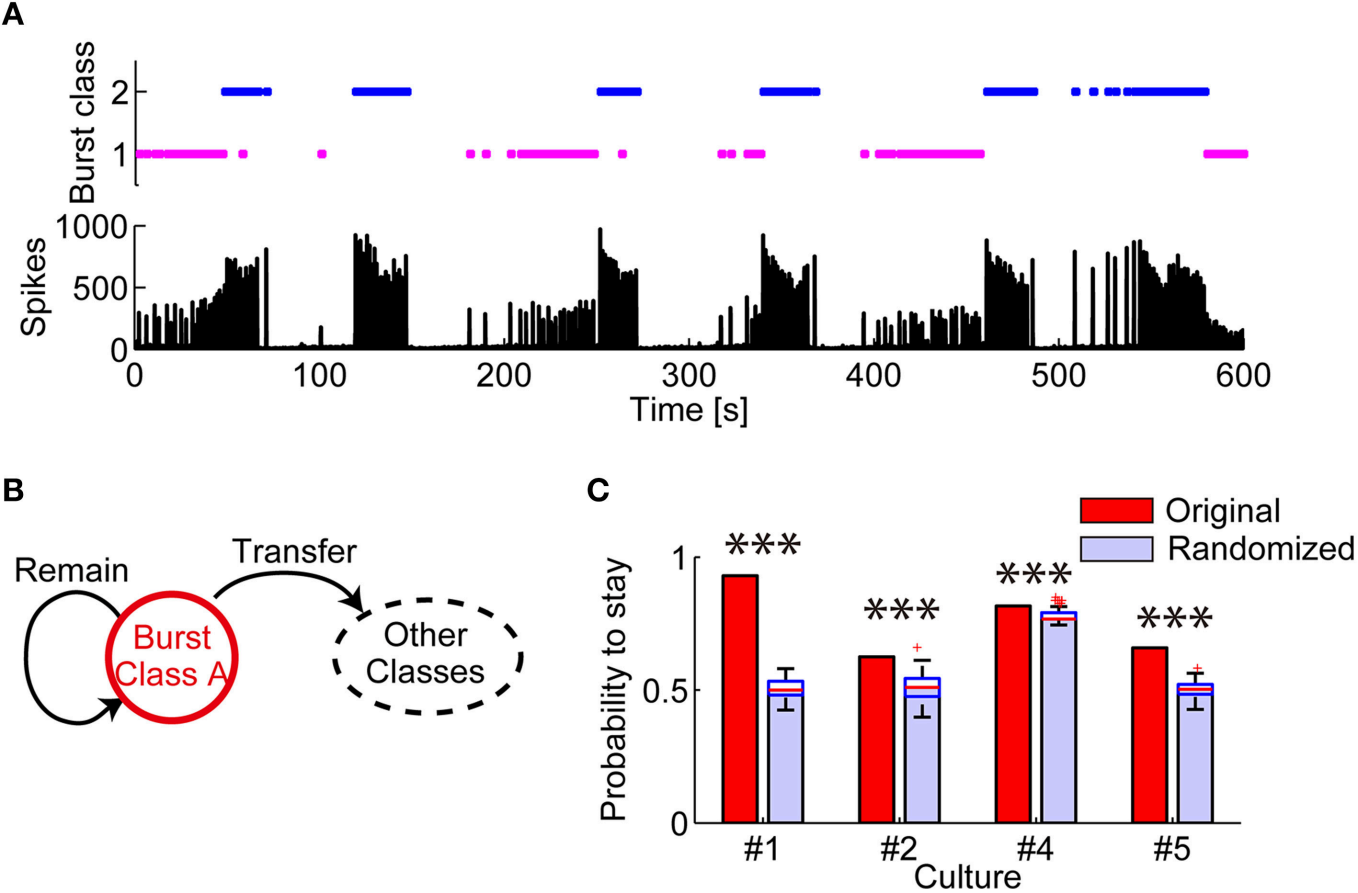
**Evaluation of consecutiveness in appearance of burst spatiotemporal patterns classes**. **(A)** (Top) Periods in which each class of bursts appeared. (Bottom) Number of array-wide spikes through the whole recording. **(B)** Schematic illustration of the transition between multiple burst classes. The probability of “remaining” was evaluated. **(C)** Probability that the same class of bursts appeared in succession. Randomized data was generated by randomly shuffling original data. The red lines in randomized data indicate the median of the probabilities. The blue boxes are ranges from the 25th percentiles to the 75th percentiles. The whiskers are the ranges of the all probabilities excluding outliers (the red crosses). One-sample Wilcoxon signed-rank test. ^***^p < 0.001.

Next, temporal consecutiveness of these spatiotemporal patterns was investigated. If spatiotemporal patterns in synchronized bursts are an active representation of hidden states of a network (Buonomano and Maass, 2009), temporally neighboring bursts should exhibit similar patterns. Figure 4A shows when each class of bursts appeared through recording, clearly demonstrating that the same class of bursts appeared consecutively. The probability of remaining in the same classes of bursts was significantly higher in the experimental data than in the simulation data with randomized orders of burst classes in all the test dishes (Figures 4B,C; Culture #1: *p* = 1.578 × 10^−30^; Culture #2: *p* = 9.466 × 10^−30^; Culture #4: 2.157 × 10^−22^; Culture #5: *p* = 1.578 × 10^−30^). In other words, a network of cortical neurons tends to maintain similar burst patterns for a certain period.

**Figure 5:**
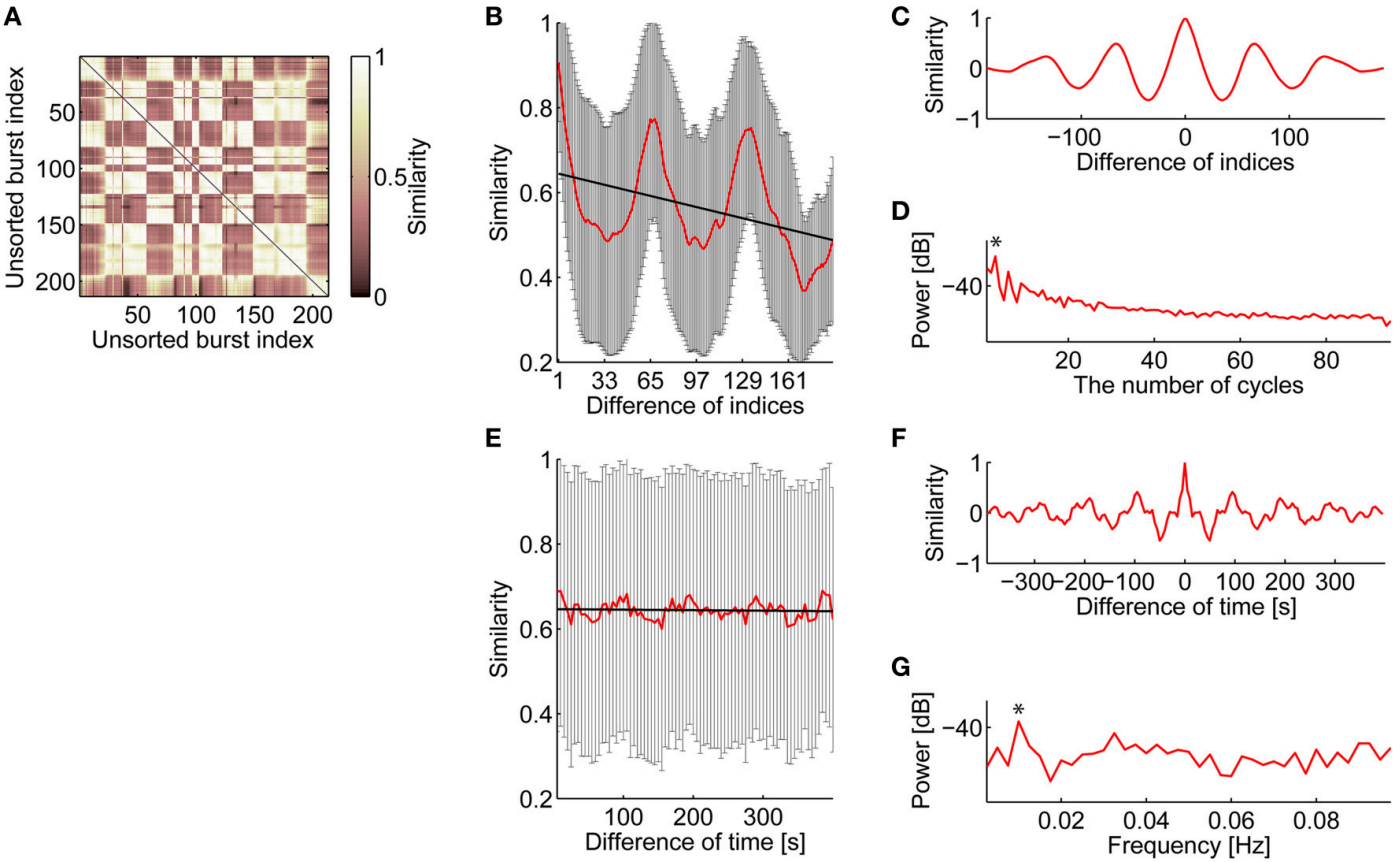
**Consecutive and periodic appearance of similar spatiotemporal patterns of bursts**. **(A)** Similarity matrix of bursts. Bursts are listed in temporal order. **(B)** Relationship between similarity and the difference of the burst indices in temporal order. The red line indicates mean similarity against the difference of burst indices in temporal order. The error bars are SD. The thick black line is a linear regression line to the mean similarity. **(C)** Autocorrelation of the mean similarity that subtracted the regression line shown in **(B)**. **(D)** Periodogram of the mean similarity that subtracted the regression line. Asterisk indicates a maximum peak. The significance level of the maximum peak was tested using Fisher's g-statistic. **(E–G)**, The same analysis as shown in **(B**–**D)**, respectively, about relationship between similarity and the difference of burst appearance time.

The consecutiveness in spatiotemporal patterns was also supported by the unsorted similarity matrix of bursts shown in Figure 5A. Similar clusters along the diagonal in the similarity matrix indicate that similar spatiotemporal patterns appeared in succession. Figure 5B illustrates the relationship between similarity of spatiotemporal patterns and the difference in burst appearance indices. Interestingly, not only did neighbor bursts share highly similar spatiotemporal patterns, but also the similar pairs of bursts seemed to appear periodically. From this observation, it was postulated that the combination of consecutiveness and periodicity can account for the burst similarity depending on the difference of burst appearance indices.

After subtracting the linear regression line, normalized autocorrelation of the similarity function (Figure 5B) was computed to visualize periodicity; then, clear periodicity was found in the autocorrelogram (Figure 5C). Fisher's g-statistic was calculated from the periodogram of the residual similarity function to evaluate the significance level of the largest component of the periodicity (Figure 5D; Wichert et al., 2003); Figure 5D indicates that similarity fluctuation in Figure 5B had 3 cycles, corresponding to 64.0 length of cycle. All cultures showed significant (*p* < 0.001) periodicity (Culture #1, *p* = 1.568 × 10^−43^; Culture #2, *p* = 1.222 × 10^−5^; Culture #4, *p* = 2.270 × 10^−3^; Culture #5, *p* = 4.829 × 10^−7^), while the length of the cycle varied between cultures (Culture #1, 64.0; Culture #2, 27.7; Culture #4, 66.0; Culture #5, 38.6). The same analysis was tested against the relationship between similarity of spatiotemporal patterns and the difference in their appearance time (Figures 5E–G). A mean interval of 64 bursts, i.e., a length of the cycle in Figure 5B, corresponds to 195.1 (±26.0, SD) s, which appeared as a peak in Figures 5E,F. All cultures except one showed significant (*p* < 0.001) periodicity (Culture #1, *p* = 3.492 × 10^−8^; Culture #2, *p* = 1.483 × 10^−8^; Culture #4, *p* = 0.1338; Culture #5, *p* = 3.492 × 10^−8^). Frequency of the similarity functions ranged between 0.01 and 0.02 Hz (Culture #1, 0.01 Hz; Culture #2, 0.175 Hz; Culture #5, 0.01 Hz).

**Figure 6:**
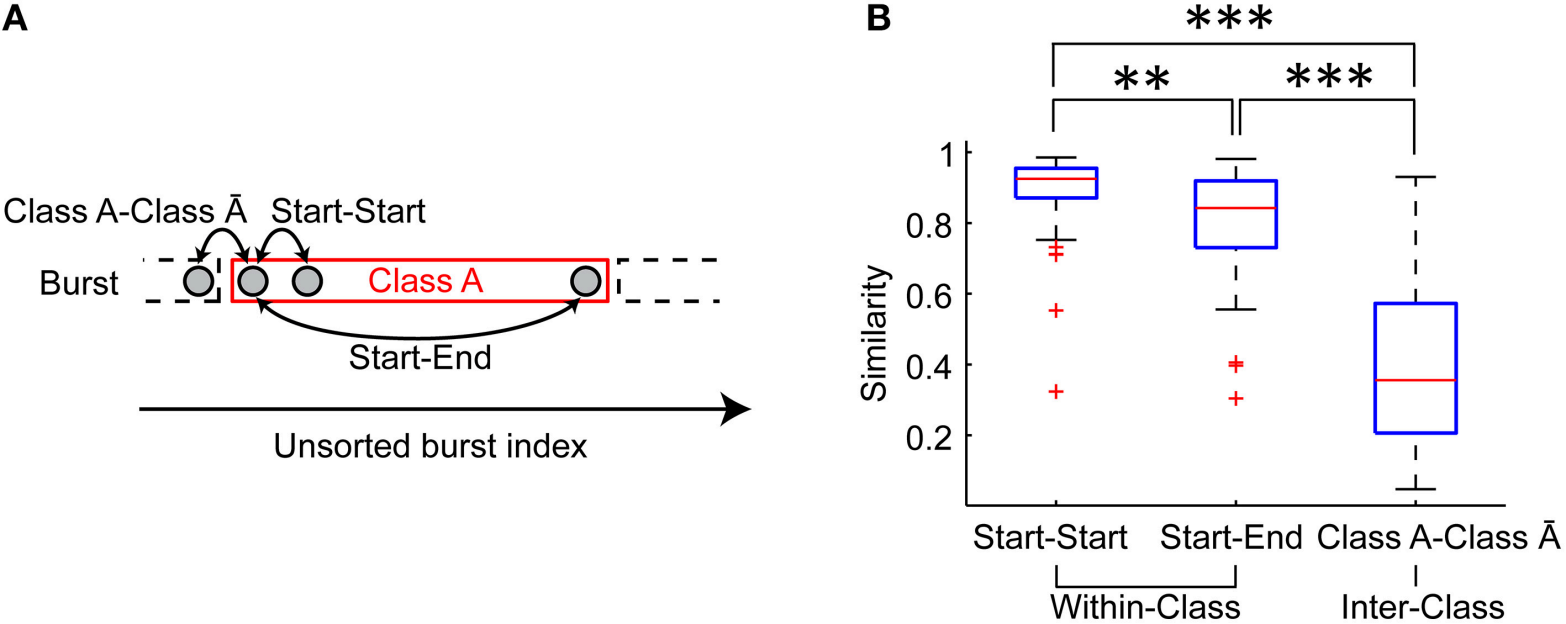
**Variation of spatiotemporal patterns during a period of the same class burst. (A)** Similarity of the first two bursts in a given period (Start-Start pair), that of the burst pair at the start and end of a given period (Start-End pair) and that of the successive burst pair at the transition of the period (Class A-Class Ā pair) were evaluated. **(B)** Similarity of Start-Start pair, Start-End pair and Class A-Class Ā pair. The red lines indicate the median of the similarities. The blue boxes are ranges from the 25th percentiles to the 75th percentiles. The whiskers are the ranges of the all similarity excluding outliers (the red crosses). Wilcoxon signed-rank test. ^**^p < 0.01. ^***^p < 0.001.

Figure 5B indicates that spatiotemporal patterns may not be homogeneous during a period of the same class bursts. To address this possibility, similarity of bursts was quantified in the first two bursts in a given period (Start-Start), in the burst pairs at the start and end of a given period (Start-End), and in the successive burst pair at the transition of the period (Class A-Class Ā) (Figure 6A). Consequently, the Start pair exhibited slightly but significantly higher similarity than the Start-End pair (*p* = 0.003), while both the Start pair and the Start-End pair exhibited significantly higher similarity than the Class A-Class Ā pair (*p* = 6.383 × 10^−7^, *p* = 3.858 × 10^−6^, respectively) (Figure 6B). Thus, spatiotemporal patterns of the same burst class gradually change with time, yet this within-class fluctuation is much smaller than the abrupt change at the transition of the burst classes.

Consequently, these results demonstrate that spatiotemporal patterns of bursts were not stochastically generated; rather, they were consecutively and periodically generated. Our results support hypothetical ideas that spatiotemporal patterns of bursts depend on hidden internal states of the network (Buonomano and Maass, 2009), and that the internal states spontaneously and recursively fluctuate between multiple “metastable” states (Durstewitz and Deco, 2007; Mazzucato et al., 2015).

## Discussion

**Figure 7:**
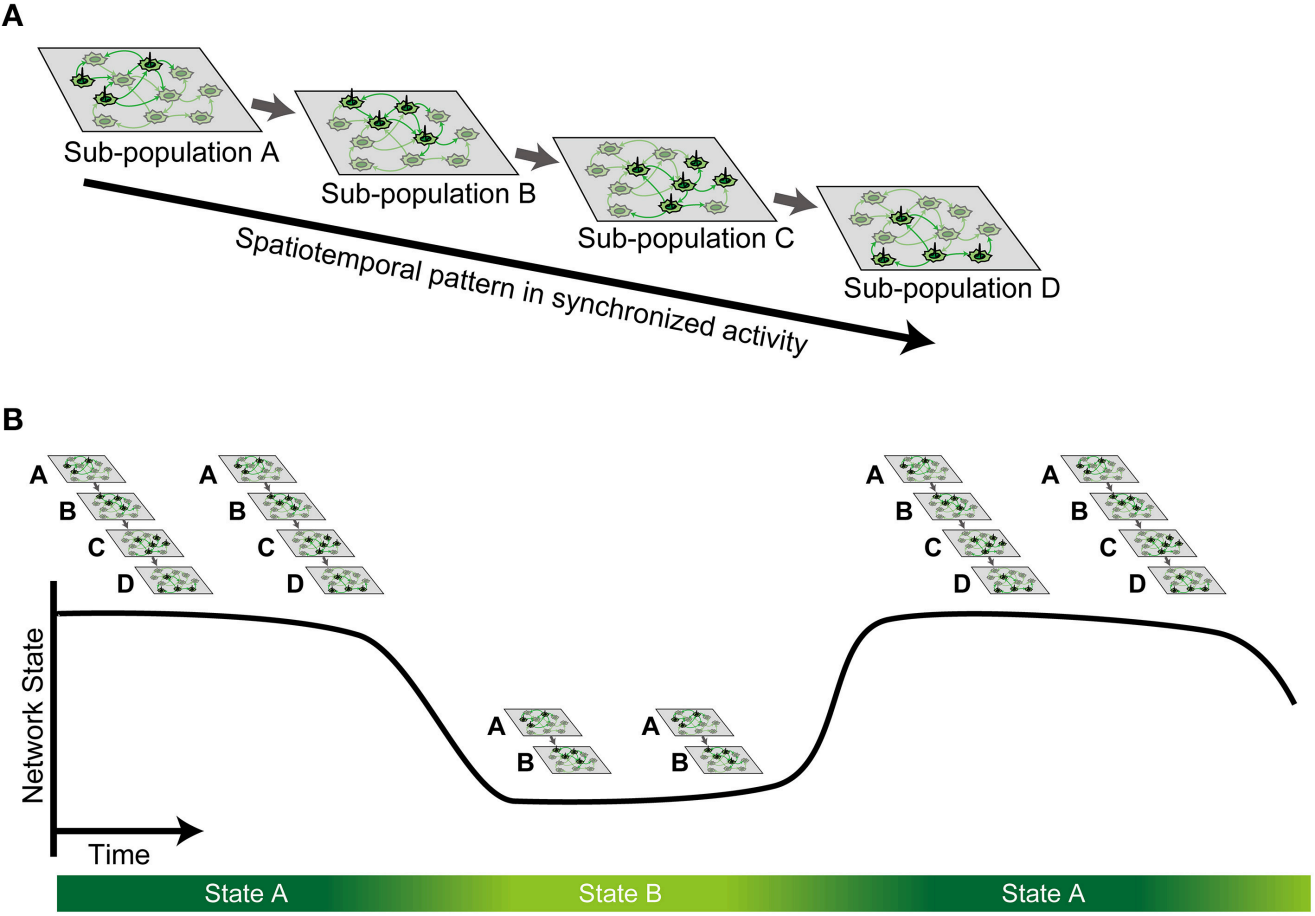
**Schematic illustration of the hypothesis**. **(A)** Stable spatiotemporal patterns observed in synchronized spontaneous activity are generated by sequential activation of neuronal sub-population. **(B)** Such sequential activation of sub-population is state-dependent, whereby multiple metastable states can be defined as a finite continuous period.

By combining high-resolution measurement with a 4096-channel CMOS MEA and dimensionality reduction with NMF, we investigated synchronized bursts of dissociated cortical neurons at approximately 3 weeks *in vitro*. We found that bursts had a repertoire of repeating spatiotemporal patterns, and different patterns shared a partially similar sequence of sub-population. These findings support the idea of propagation of neuronal sub-populations during synchronized activity (Figure 7A; Abeles, 1991; Ikegaya et al., 2004; Eytan and Marom, 2006). Furthermore, we found that similar spatiotemporal patterns tended to appear successively and periodically, suggesting state-dependent fluctuation of propagation (Figure 7B), which is overlooked in existing literature. Thus, such a state-dependent property within the sequential structure is a plausible neural substrate for performing a repertoire of stable patterns during synchronized activity.

### Methodological significances

CMOS MEA is an emerging platform for capturing electrophysiological activity of neuronal networks. It is analogous to a movie with a spatial resolution at a cellular level and temporal resolution at a single action potential (Berdondini et al., 2009). Only a small population of neurons exhibit high activity, which plausibly play crucial roles in the network (Wohrer et al., 2013); overlooking these neurons may lead to misinterpretation of results in our experiments. Thus, to avoid such sampling bias, cellular-level spatial resolution is required in the measurement (Panas et al., 2015). Furthermore, because typical burst activities of our interests last only for a few 100 ms (Eytan and Marom, 2006), the temporal resolution should be on the order of ms to appropriately characterize the pattern in bursts. CMOS MEA is the only measurement device available that meets both of these spatial and temporal requirements.

The dimensions of CMOS MEA data are inherently much higher than those of the functional SPPs (Baruchi and Ben-Jacob, 2004) of our interests. Appropriate dimensionality reduction is therefore helpful in identifying functional patterns. In the present study, we employed NMF to identify stably co-activated neuronal sub-populations. Originally, NMF was developed to extract characteristic parts, such as an eye, nose, and mouth, from facial pictures (Lee and Seung, 1999). The practical advantages of NMF are that there is no need for pre-processing (Peyrache et al., 2009; Lopes-dos-Santos et al., 2013), and that non-negative components extracted from the spiking activity patterns are intuitively interpretable, just like the facial parts in facial pictures (Lee and Seung, 1999).

### Repeating spatiotemporal patterns in synchronized spontaneous activity

Consistent with the present results, previous studies showed that cortical cultures have a repertoire of repeating spatiotemporal patterns (Segev et al., 2004; Madhavan et al., 2007; Rolston et al., 2007). A bimodal burst-peak-amplitude distribution and bimodal spatiotemporal patterns of bursts were typically observed in our experiments. In terms of burst peak amplitude, developed cultures (around 21 DIV) exhibited a bimodal distribution, whereas young cultures showed a uni-modal distribution (Eytan and Marom, 2006; Wagenaar et al., 2006b; Madhavan et al., 2007). Similarity-based clustering also demonstrated that synchronized bursts could be classified into a few patterns (Segev et al., 2004); i.e., small and large bursts (Madhavan et al., 2007). Our results extend these findings in that both large and small bursts share similar activation sequences of sub-populations.

### State-dependency and spatiotemporal patterns

Our results demonstrate the spontaneous itinerancy between different classes of spatiotemporal activity, suggesting that metastable states exist in innately isolated neuronal networks *in vitro*. Some intermediate states may also exist because similarity of burst patterns fluctuated continuously within a given state. Similar spontaneous transitions between metastable states were recently reported in the gustatory cortex *in vivo* (Mazzucato et al., 2015). Cortical activities were also characterized as two extreme states, i.e., desynchronized and synchronized states, with continuum of intermediate states (Harris and Thiele, 2011). These metastable states have a time scale of seconds or minutes. Thus, they are different from previously described metastable states in dissociated networks through development, which have a time scale of weeks or months and transit only unidirectionally (Pu et al., 2013).

A stable activity state of a neuronal network has been often mentioned as an attractor (Cossart et al., 2003; Wagenaar et al., 2006a). Classically, an attractor in the neural network was postulated as a memory of specific information (Hopfield, 1982). The classical attractor networks, however, are biologically implausible because the number of attractors is limited compared to the capacity of information (Maass et al., 2002) because the converging time into attractors (Maass et al., 2002; Rabinovich et al., 2008) and the effect of spontaneous activity (Kurikawa and Kaneko, 2015) are not consistent with experimental observation. Therefore, according to recent studies, it is more biologically plausible that transient metastable dynamics dominate neuronal activity (Durstewitz and Deco, 2007; Rabinovich et al., 2008).

Usually, repeating spatiotemporal activity is apprehended only as a visible sign of a metastable state (Haldeman and Beggs, 2005; Mazzucato et al., 2015) during which cellular and synaptic properties—e.g., the effect of short-term plasticity, slow inhibitory post-synaptic potentials, NMDA channel kinetics, etc.—are lasting, forming a so-called “hidden state” of neuronal networks (Buonomano and Maass, 2009). Such hidden states could account for consecutive appearances of similar bursts and can be considered an internal memory of a neuronal network. This internal memory is likely stronger than the short-term memory of external events, which is easily broken by bursts; i.e., internal memory (Dranias et al., 2013, 2015; Ju et al., 2015). Inhibitory interneurons may significantly contribute to selection of spatiotemporal patterns (Sasaki et al., 2014), depending on such hidden states.

In addition, our results demonstrate that spontaneous bursts may induce state transitions. Similarly, co-activation of some neurons trigger a state transition in the gustatory cortex (Mazzucato et al., 2015). These findings suggest that the hidden states in a neuronal network dominate spatiotemporal patterns of spontaneous activities, which in turn modulate the hidden states. Such an interaction between the hidden states and spontaneous bursts is a possible underlying mechanism of the metastable activity in neuronal networks.

### Sequential propagation structures during synchrony

Our results imply that multiple spatiotemporal patterns are generated by a common stable propagation structure in the network (Raichman and Ben-Jacob, 2008). Signal transmission within such a stable structure might therefore depend on the hidden states. This conceptual framework of sequential structure is compatible with previous findings of a small group of “leader neurons,” which activate at burst initiation, and hierarchical structures in the dissociated neuronal networks (Eytan and Marom, 2006; Ham et al., 2008). Our results are also consistent with *in vivo* experiments in that activity bursts across states have similar spatiotemporal patterns (Luczak et al., 2013). Taken altogether, such a modified model of synfire chain with state-dependent fluctuation can account for both stability (Eytan and Marom, 2006; Panas et al., 2015) and multiple pattern generation (Segev et al., 2004; Madhavan et al., 2007; Rolston et al., 2007) in synchronized activities.

Existing models mostly overlooked state-dependent property to account for the variety of spatiotemporal patterns in the neuronal network. For example, a branching process is one of the convincing models (Beggs and Plenz, 2003). It demonstrates that cortical networks *in vitro* have metastable states, and that the critical branching process maximizes the number of metastable states (Haldeman and Beggs, 2005). Fixed propagation probabilities between neurons are postulated in these models, and spatiotemporal patterns are generated stochastically. However, this model is inconsistent with our finding that each spatiotemporal pattern does not randomly emerge; instead, it is repeated in a temporally consecutive manner. Further modeling with state-dependent properties would be one of the future directions.

### Spontaneous spatiotemporal patterns *in vivo*

The repeating spatiotemporal patterns in spontaneous activities have been observed not only *in vitro* (Beggs and Plenz, 2004; Ikegaya et al., 2004), but also *in vivo* (Luczak et al., 2007). Neuronal networks may transmit information as “neuronal packets” (Luczak et al., 2013, 2015), i.e., activity of neuronal sub-population, generating such stable patterns, which is consistent with our results. Memory replay in the hippocampus is extensively studied as a possible mechanism of memory consolidation during sleep (Lee and Wilson, 2002) and memory retrieval at awaking immobility (Takahashi, 2015; Villette et al., 2015). In the sensory cortex, Luczak et al. found similarity between spontaneous patterns and evoked ones. They hypothesized that a repertoire of evoked responses is a fraction of a spontaneous repertoire (Luczak et al., 2009). Spontaneous activity might be considered a prior distribution of sensory inputs (Berkes et al., 2011).

Nevertheless, in the network that experiences no external inputs, we demonstrated that repeating spatiotemporal patterns emerge in a state-dependent manner. Such network could have similar functional structures during spontaneous activity and stimulus-evoked activity (Pirino et al., 2015). Future extensive studies in both experimental and theoretical approaches are required to elucidate the functions and mechanisms of these spontaneous properties.

## Author contributions

YY, RK, and HT designed research; YY performed research, composed programs and analyzed data; YY, RK, and HT wrote the paper.

### Conflict of interest statement

The authors declare that the research was conducted in the absence of any commercial or financial relationships that could be construed as a potential conflict of interest.

## Footnotes

^1^https://github.com/audiofilter/nmflib

## Abbreviations

BFM: Burst feature matrix
SPP: Sub-population pattern
SPAW: Sub-population activation weight.
